# An Interactive Game with Virtual Reality Immersion to Improve Cultural Sensitivity in Health Care

**DOI:** 10.1089/heq.2021.0128

**Published:** 2022-03-03

**Authors:** Paul J. Hershberger, Yong Pei, Timothy N. Crawford, Sabrina M. Neeley, Thomas Wischgoll, Dixit B. Patel, Miteshkumar M. Vasoya, Angie Castle, Sankalp Mishra, Lahari Surapaneni, Aman A. Pogaku, Aishwarya Bositty, Todd Pavlack

**Affiliations:** ^1^ Department of Family Medicine, Wright State University Boonshoft School of Medicine, Dayton, Ohio, USA.; ^2^ Department of Computer Science and Engineering, Wright State University College of Engineering and Computer Science, Dayton, Ohio, USA.; ^3^ Department of Population and Public Health Sciences and Department of Family Medicine, Wright State University Boonshoft School of Medicine, Dayton, Ohio, USA.; ^4^ School of Education and Health Sciences, University of Dayton, Dayton, Ohio, USA.; ^5^ Manager Distance Learning and Instructional Designer, Wright State University, Dayton, Ohio, USA.

**Keywords:** bias, prejudice, social determinants of health, simulation, virtual reality

## Abstract

**Purpose:**

Biased perceptions of individuals who are not part of one's in-groups tend to be negative and habitual. Because health care professionals are no less susceptible to biases than are others, the adverse impact of biases on marginalized populations in health care warrants continued attention and amelioration.

**Method:**

Two characters, a Syrian refugee with limited English proficiency and a black pregnant woman with a history of opioid use disorder, were developed for an online training simulation that includes an interactive life course experience focused on social determinants of health, and a clinical encounter in a community health center utilizing virtual reality immersion. Pre- and post-survey data were obtained from 158 health professionals who completed the simulation.

**Results:**

Post-simulation data indicated increased feelings of compassion toward the patient and decreased expectations about how difficult future encounters with the patient would be. With respect to attribution, after the simulation participants were less inclined to view the patient as primarily responsible for their situation, suggesting less impact of the fundamental attribution error.

**Conclusion:**

This training simulation aimed to utilize components of evidence-based prejudice habit breaking interventions, such as learning more about an individual's life experience to help minimize filling in gaps with stereotyped assumptions. Although training simulations cannot fully replicate or replace the advantages that come with real-world experience, they can heighten awareness in the increase of increasing the cultural sensitivity of clinicians in health care professions for improving health equity.

## Introduction

Biases can be understood as a byproduct of the shortcuts that human beings automatically and necessarily take in the processing of information.^1^ Importantly, biased perceptions about other people, particularly those not a part of one's in-groups, become habitual and are often negative.^2^ The fundamental attribution error refers to the tendency to attribute the behavior of others to dispositional factors rather than situational factors,^3^ a proclivity that exacerbates group stereotypes. Whether explicit or implicit, biases toward outgroups can overtly or covertly affect behavior toward others.

Health care professionals are no less susceptible to biases than are others.^4^ While the effect of biases on the care provided to patients may not be readily apparent, there is enough evidence to conclude that the negative impact of explicit and implicit biases on marginalized populations in health care warrants attention and amelioration.^5,6^

The most promising efforts to minimize the impact of implicit biases and prejudicial beliefs on behavior have been based upon the prejudice habit model that views implicit biases as habits that can be modified with sufficient awareness, motivation, and effort.^7^ Prejudice habit-breaking interventions have yielded both immediate and longer term (e.g., up to 2 years later) benefits, in spite of how resistant prejudicial biases can be to change.^8^

These evidence-based interventions typically include one or more of the following strategies: stereotype replacement, individuation, perspective taking, counter stereotypic imaging, and increasing opportunities for contact with out-group members.^9,10^ The impact of implicit bias on disparities in health care may be lessened if physicians recognize their own biases and practice the skills of perspective taking and individuation.^11–13^

Components of this habit-breaking intervention formed the basis for a simulation named “SDOH Sim” that we developed as part of the Medicaid Equity Simulation (MES) project funded by the Ohio Medicaid Technical Assistance and Policy Program (MEDTAPP). The primary goal of the MES was to create training experiences that included virtual or augmented reality features aimed to decrease the impact of implicit bias on the part of clinicians treating patients receiving Ohio Medicaid benefits (https://grc.osu.edu/projects/MEDTAPP/MES).

Our training simulation utilizes an augmented, modified, and digital version of the Life Course game, an interactive training tool for health professionals designed for understanding the social determinants of health. Participants receive birth certificates at the start of the game that identify social and biologically based factors that help determine their course and challenges in life. These factors convey advantage and/or disadvantage as each participant proceeds through life and experiences significant events, such as losing a job or developing diabetes. Each roll of the dice identifies additional risk and/or protective factors that either push down or lift up their overall health trajectory and life course.

Originally developed and produced in 2008 by CityMatCH, in 2017 Wright State University adapted the game to a digital and online version so that numerous medical students could play the game simultaneously. For the MES project, the Life Course game provided a format conducive to emphasis on the impact of the social determinants of health. We created two new characters for the MES project: (1) a Syrian refugee with limited English proficiency, and (2) a black pregnant woman with a history of opioid use disorder. These characters were among those suggested in the request for proposals from the MES project, and included features for which members of the project team had clinical experience.

A health professional participating in the simulation is randomly assigned to vicariously experience the life course of one of these disadvantaged individuals. A brief description of the individual is provided. The Life Course game portion of the simulation includes a companion character, that is, a preprogrammed and corresponding digital player who has higher educational attainment than does the disadvantaged individual.

**FIG. 1.**
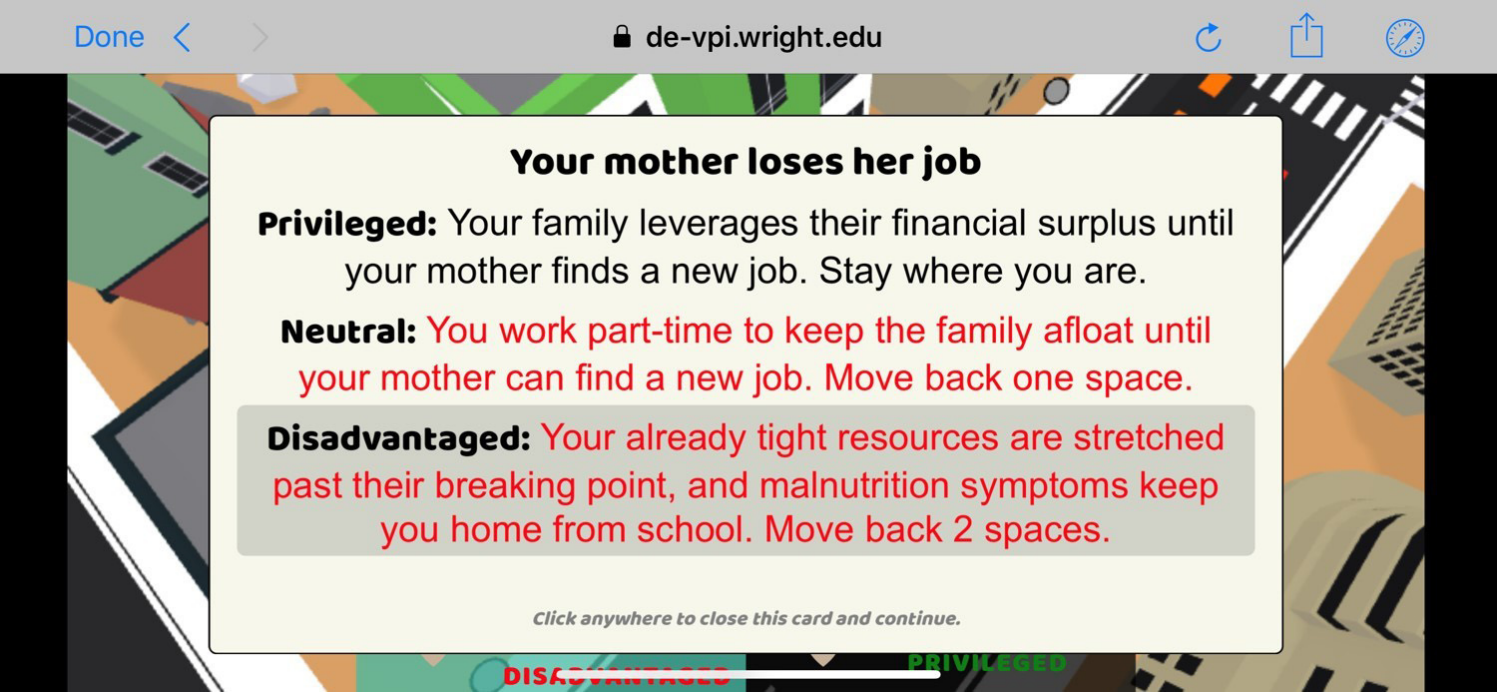
Example of differential impact of a life event based upon socioeconomic status.

As both the disadvantaged and advantaged characters proceed through their life experiences, the impact of educational level and socioeconomic factors on life events is highlighted (Fig. 1). The training participant progresses through the game from the perspective of their assigned character from birth to approximately age 30, and witnesses the corresponding life course of the advantaged individual.

**FIG. 2.**
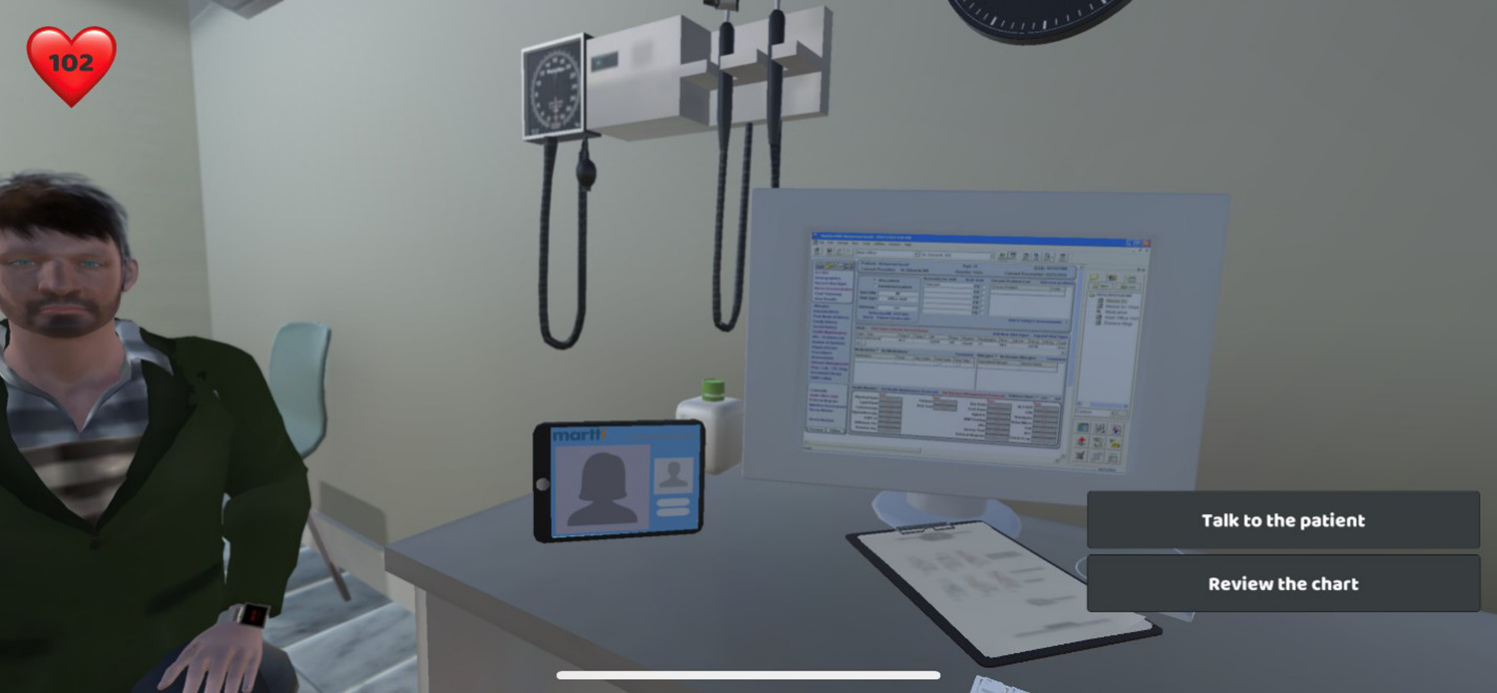
Image of virtual reality immersion from the provider perspective.

At age 30, the Life Course game portion of the simulation ends and the immersive virtual reality (VR) scenario begins when the individual presents for care at a community health center. The VR includes four elements of the health center visit: (1) preparation for and transportation to the appointment; (2) check-in at the health center; (3) interaction with the clinician/provider (this phase is from the perspective of the provider rather than the patient) (Fig. 2); and (4) filling prescriptions in the refugee case and attempting to obtain medication-assisted treatment in the pregnant woman case. The overall aim of the simulation is to enhance provider awareness of the importance of understanding the individual perspective and experiences of members of groups for whom implicit biases are likely present.

The training simulation can be completed remotely on a tablet or smartphone in any location, as long as there is internet connection, and requires ∼25–30 min. The simulation utilizes a mobile application that is available on the App Store (Apple iOS) and Google Play (Android).

## Method

### Participants

Health professionals were recruited to participate in the simulation with several strategies. These included reaching out to area health organizations, such as community health centers, and offering to do in-person training sessions that included completion of the simulation. We also developed brochures that were distributed to staff in other health organizations that encouraged completion of the simulation on their own time. Of the 364 clinical and nonclinical health professionals who have done the simulation, 158 individuals completed pre- and post-simulation questions. Demographic characteristics of these 158 participants are summarized in Table 1. This study was determined to be exempt by the Institutional Review Board of Wright State University.

**Table 1.** Demographics for Simulation Participants (*N*=158)

Variables	*n* (%)
Age, mean (SD)	32.6 (12.9)
Sex	
Female	119 (75.8)
Male	38 (24.2)
Race	
White	122 (77.2)
Black	9 (5.7)
Asian	16 (10.1)
Hispanic	7 (4.4)
Other	4 (2.5)
Scenario	
Syrian refugee	105 (66.5)
Pregnant mother	52 (32.9)
Profession type^*^	
Nonclinical personnel	55 (35.0)
Clinical non-Medicaid provider	82 (52.2)
Clinical Medicaid provider	20 (12.7)
I work in a	
Behavioral Health Setting	3 (2.7)
Federally Qualified Health Center	37 (33.3)
Healthcare System Affiliated Clinic	5 (4.5)
Hospital	13 (11.7)
Private practice	3 (2.7)
Other	50 (45.1)
Percentage of Medicaid patients that you serve	
>30%	53 (47.8)
≤30%	19 (17.1)
Practice does not see Medicaid patients	39 (35.1)

^*^ 32.9% (*n*=52) were physicians.

Missing values are not included in calculation of percentages.

SD, standard deviation.

### Measures

Nine pre- and post-training questions were written by the authors and are included in the simulation itself. No reliability or validity studies have been done with these survey items. Survey brevity was emphasized along with a goal to include items addressing emotion, expectations, attributions, and motivation. Items 1–3 assess emotional reactions to the patient, items 4–5 measure expectations about the encounter with the patient, items 6–7 measure the presence of the fundamental attribution error, and items 8–9 identify internal and external motivation to respond without prejudice.

A bias scale score was created by summing up the nine items, with items 3, 7, and 9 being reverse scored. The scores ranged from 9 to 45 with higher scores suggesting more bias (Cronbach's alpha=0.62). There are three additional post-training questions to obtain feedback about the training experience itself. Questions are included in Table 2.

**Table 2.** Pre- and Post-Test Questions Among Participants Responding to both Pre- and Post-Test Questions (*N*=158)

	Presim, mean (SD)	Postsim, mean (SD)	Difference,^a^mean (SD)	*p* ^b^
With respect to having this individual as my next patient, the amount of ANXIETY I feel is: (1=low to 5=high)	2.4 (1.2)	2.3 (1.2)	0.11 (1.1)	0.19
With respect to having this individual as my next patient, the amount of FRUSTRATION I feel is: (1=low to 5=high)	1.9 (1.1)	2.1 (1.1)	−0.18 (1.1)	0.05
With respect to having this individual as my next patient, the amount of COMPASSION I feel is^a^: (1=low to 5=high)	4.2 (0.9)	4.5 (0.9)	−0.28 (1.1)	0.001
If given a choice, instead of this patient I would prefer to see a different patient for routine follow-up of a chronic health problem such as hypertension. (1=strongly disagree to 5=strongly agree)	2.3 (1.2)	2.1 (1.2)	0.22 (1.0)	0.008
I expect that my encounter with this patient will be: (1=easy to 5=difficult)	3.1 (1.1)	2.9 (1.0)	0.26 (1.2)	0.009
I believe that this patient is largely responsible for being in their current circumstances. (1=strongly disagree to 5=strongly agree)	1.9 (1.0)	1.7 (0.9)	0.27 (0.8)	0.0001
I believe that the circumstances in which this patient finds themself are largely beyond their control.^a^ (1=strongly disagree to 5=strongly agree)	3.6 (1.0)	4.0 (1.2)	−0.37 (1.2)	0.0001
I try to hide any negative thoughts about patients like this to avoid negative reactions from others. (1=never to 5=always)	3.7 (1.3)	4.0 (1.3)	−0.25 (1.0)	0.002
I attempt to act in nonprejudiced ways toward patients like this because it is personally important to me.^a^ (1=never to 5=always)	4.7 (0.6)	4.7 (0.7)	−0.1 (0.7)	0.37
I believe going through this training experience will help me decrease any negative biases I may have toward patients like this. (1=strongly disagree to 5=strongly agree)		3.8 (1.1)		
I believe going through this training experience will help me be a more understanding health professional. (1=strongly disagree to 5=strongly agree)		3.9 (1.1)		
This activity was an effective learning platform: (1=strongly disagree to 5=strongly agree)		3.8 (1.2)		
Bias Scale Score	20.9 (5.1)	19.7 (5.0)	0.268	0.0009

^a^ Items were reverse scored to create the bias scores.

^b^ Tests conducted were paired *t*-tests.

Mean difference is calculated as pre–post.

### Procedure

Upon accessing the simulation on a tablet or smartphone, participants initially respond to several demographic questions. Subsequently, a brief video from the VR immersion introduces the participant to “their next patient.” The participant then responds to the nine pre-training questions. Next, the simulation proceeds into the life course portion of the experience. Upon completion of the game portion and the VR immersion components of the simulation, post-training questions are presented to the participant.

### Data analytic approach

Descriptive statistics were calculated to describe the study participants with means and standard deviations for continuous and ordinal variables and frequencies and percentages for categorical variables. To examine changes in the pre- and post-training questions, paired *t*-tests were conducted for individuals with responses to both the pre- and post-training questions. Standardized mean differences (SMDs) (mean difference/standard deviation) were calculated to observe the size of the effects. SMDs of 0.2, 0.5, and 0.8 are considered small, moderate, and large effect sizes, respectively. Differences in responses were calculated as pre–post.

To assess differences in bias questions, we stratified the results by profession group status (clinical and nonclinical) and scenario (Syrian refugee and pregnant mother). A secondary analysis was conducted to compare pre-simulation questions between those who completed post-simulation questions and those who did not. Independent *t*-tests and chi-square tests were used for continuous and categorical variables, respectively. All data were analyzed using SAS version 9.4 (Cary, NC) and *p*-values <0.05 were regarded as statistically significant.

## Results

Table 1 provides the characteristics of the simulation participants who completed both pre- and post-simulation questions overall and by profession group (*N*=158). The average age of the participants was 32.6±12.9 years. The majority of the participants were female (75.8%), white (77.2%), and completed the Syrian refugee simulation (66.5%).

Almost one-third of participants were clinical personnel (64.9%). A third of the clinical personnel were nurses and ∼51% of the clinical personnel were physicians. The clinical personnel were older compared with the nonclinical personnel (35.8 years vs. 26.3 years) and were more likely to be male compared with the nonclinical personnel (31.7% vs. 10.9%). The clinical personnel were more likely to work in a federally qualified health center compared with the nonclinical personnel (52.5% vs. 11.8%). Nonclinical personnel included managers and administrators, although data on specific positions were not obtained.

The Syrian refugee case was developed and deployed before the pregnant women case. Therefore, there were 93 participants that completed the simulation when the Syrian refugee case was the only one available. Subsequently, the number of participants completing the two cases was nearly equal. This explains why the distribution is not similar. Also, ∼60% of those in the pregnant mother case did not have complete data (pre and post) compared with 55% of those in the Syrian refugee case.

Table 2 provides the responses to the nine pre- and post-simulation questions for the entire analytic sample. From pre- to post-simulation, there were significant changes on 7 of the 9 survey questions. The effects for these changes were small to moderate in size.

These included increased feelings of compassion toward the patient (SMD=−0.263, *p*=0.001), but more frustration with the anticipation of having the simulation patient as one's next patient (SMD=−0.157, *p*=0.05). Respondents were less likely to indicate a preference for seeing a different patient for a routine follow-up appointment rather than the patient in the simulation (SMD=0.213, *p*=0.008), and there was a decrease in expectation about how difficult the encounter with the simulation patient would be (SMD=0.213, *p*=0.009). Also, there was a decrease in the degree to which the individual was seen as largely responsible for her or his situation (SMD=0.316, *p*=0.0001), and an increase in seeing the individual's circumstances as beyond their control (SMD=−0.309, *p*=0.0001), representing some decreased expression of the fundamental attribution error.

The increase in a tendency to try to hide negative thoughts about patients to avoid negative reactions from others was also significant (SMD=−0.256, *p*=0.002). There was a decrease in bias overall among the participants (SMD=0.268, *p*=0.0009), however, the bias scale score should be interpreted with caution as the Cronbach's alpha suggests low internal consistency and reliability, likely due to the aim of assessing several factors with only nine items.

Table 3 shows results of analyses of pre- and post-simulation survey responses in which nonclinical and clinical participants were separated. For the clinical personnel participants, there were significant increases in the compassion felt for the patient (SMD=−0.290, *p*=0.004) and decreases in the level of difficulty expected for the patient encounter (SMD=0.245, *p*=0.02). Significant changes for both groups were the two questions related to the fundamental attribution error.

**Table 3.** Pre- and Post-Test Questions by Clinical Status (*N*=158)

	Nonclinical (*n*=55)			Clinical (*n*=102)		
	Presim, mean (SD)	Postsim, mean (SD)	Difference, mean (SD)	Presim, mean (SD)	Postsim, mean (SD)	Difference, mean (SD)
With respect to having this individual as my next patient, the amount of ANXIETY I feel is: (1=low to 5=high)	2.5 (1.2)	2.4 (1.2)	0.04 (1.4)	2.4 (1.3)	2.3 (1.1)	0.14 (0.9)
With respect to having this individual as my next patient, the amount of FRUSTRATION I feel is: (1=low to 5=high)	1.8 (1.0)	2.1 (1.2)	−0.35 (1.3)	1.9 (1.1)	2.0 (1.1)	−0.10 (1.1)
With respect to having this individual as my next patient, the amount of COMPASSION I feel is^a^: (1=low to 5=high)	4.3 (0.9)	4.4 (0.8)	−0.16 (0.8)	4.1 (0.9)	4.5 (0.9)	−0.34 (1.2)^*^
If given a choice, instead of this patient I would prefer to see a different patient for routine follow-up of a chronic health problem such as hypertension. (1=strongly disagree to 5=strongly agree)	2.3 (1.2)	2.0 (1.2)	0.25 (0.9)^*^	2.4 (1.3)	2.2 (1.2)	0.19 (1.1)
I expect that my encounter with this patient will be: (1=easy to 5=difficult)	2.9 (1.1)	2.7 (1.1)	0.20 (1.2)	3.3 (1.1)	3.0 (0.9)	0.30 (1.3)^*^
I believe that this patient is largely responsible for being in their current circumstances. (1=strongly disagree to 5=strongly agree)	1.9 (0.9)	1.5 (0.8)	0.42 (0.8)^*^	1.9 (1.0)	1.7 (1.0)	0.18 (0.9)^*^
I believe that the circumstances in which this patient finds themself are largely beyond their control.^a^ (1=strongly disagree to 5=strongly agree)	3.7 (1.1)	4.1 (1.1)	−0.35 (1.19)^*^	3.6 (1.0)	4.0 (1.2)	−0.39 (1.2)^*^
I try to hide any negative thoughts about patients like this to avoid negative reactions from others. (1=never to 5=always)	4.0 (1.3)	4.1 (1.3)	−0.18 (0.9)	3.6 (1.3)	3.9 (1.2)	−0.29 (1.0)^*^
I attempt to act in nonprejudiced ways toward patients like this because it is personally important to me.^a^ (1=never to 5=always)	4.8 (0.6)	4.8 (0.8)	0.02 (0.6)	4.6 (0.6)	4.7 (0.6)	−0.09 (0.8)
I believe going through this training experience will help me decrease any negative biases I may have toward patients like this. (1=strongly disagree to 5=strongly agree)		4.2 (0.9)			3.7 (1.2)	
I believe going through this training experience will help me be a more understanding health professional. (1=strongly disagree to 5=strongly agree)		4.2 (0.9)			3.8 (1.2)	
This activity was an effective learning platform: (1=strongly disagree to 5=strongly agree)		3.9 (1.0)			3.7 (1.2)	
Bias Scale Score	20.4 (4.9)	19.5 (5.4)	0.87 (4.5)	21.2 (5.2)	19.8 (4.9)	1.28 (4.2)^*^

^a^ Items were reverse scored to create the bias scores.

^*^ *p*<0.05.

Mean difference is calculated as pre–post.

Table 4 shows the results of the pre- and post-simulation responses for each scenario separately. The majority of the participants completed the Syrian refugee simulation (66.5%). For the participants with Syrian refugee case, there were significant decreases in preference for seeing a different patient (SMD=0.276, *p*=0.007) and difficulty in encounter with patient (SMD=0.360, *p*=0.0004). In addition, those participating in the Syrian refugee simulation were more likely to agree that the patient's circumstances were beyond their control, post-simulation (SMD=−0.336, *p*=0.0008). For the pregnant mother simulation, there was an increase in the amount of compassion felt among the participants (SMD=−0.312, *p*=0.03).

**Table 4.** Pre- and Post-Test Questions by Simulation Scenario (*N*=157)

	Syrian refugee (*n*=105)			Pregnant mother (*n*=52)		
	Presim, mean (SD)	Postsim, mean (SD)	Standard mean difference	Presim, mean (SD)	Postsim, Mean (SD)	Standard mean difference
With respect to having this individual as my next patient, the amount of ANXIETY I feel is: (1=low to 5=high)	2.6 (1.2)	2.4 (1.1)	0.202^*^	2.1 (1.2)	2.3 (1.2)	−0.124
With respect to having this individual as my next patient, the amount of FRUSTRATION I feel is: (1=low to 5=high)	2.1 (1.1)	2.1 (1.2)	0.024	1.5 (1.0)	2.0 (1.1)	−0.45^*^
With respect to having this individual as my next patient, the amount of COMPASSION I feel is^a^: (1=low to 5=high)	4.2 (0.8)	4.5 (0.8)	−0.237	4.0 (1.0)	4.4 (1.0)	−0.312^*^
If given a choice, instead of this patient I would prefer to see a different patient for routine follow-up of a chronic health problem such as hypertension. (1=strongly disagree to 5=strongly agree)	2.4 (1.3)	2.1 (1.2)	0.276^*^	2.2 (1.2)	2.1 (1.1)	0.078
I expect that my encounter with this patient will be: (1=easy to 5=difficult)	3.3 (1.1)	2.9 (1.0)	0.360^*^	2.8 (1.1)	2.8 (1.0)	0.015
I believe that this patient is largely responsible for being in their current circumstances. (1=strongly disagree to 5=strongly agree)	1.8 (1.0)	1.5 (1.0)	0.355	2.1 (0.9)	1.9 (0.8)	0.248
I believe that the circumstances in which this patient finds themself are largely beyond their control.^a^ (1=strongly disagree to 5=strongly agree)	3.8 (1.0)	4.2 (1.1)	−0.336^*^	3.3 (1.0)	3.6 (1.1)	−0.249
I try to hide any negative thoughts about patients like this to avoid negative reactions from others. (1=never to 5=always)	3.7 (1.3)	3.9 (1.3)	−0.190	3.8 (1.3)	4.1 (1.1)	−0.395^*^
I attempt to act in nonprejudiced ways toward patients like this because it is personally important to me.^a^ (1=never to 5=always)	4.7 (0.6)	4.7 (0.7)	0.047	4.7 (0.6)	4.8 (0.7)	−0.161
I believe going through this training experience will help me decrease any negative biases I may have toward patients like this. (1=strongly disagree to 5=strongly agree)		3.8 (1.2)			4.0 (1.1)	
I believe going through this training experience will help me be a more understanding health professional. (1=strongly disagree to 5=strongly agree)		3.9 (1.1)			4.0 (1.0)	
This activity was an effective learning platform: (1=strongly disagree to 5=strongly agree)		3.7 (1.2)			3.9 (1.2)	
Bias Scale Score	21.1 (5.1)	19.4 (5.1)	0.396^*^	20.5 (5.2)	20.5 (4.8)	0.005

^a^ Items were reverse scored to create the bias scores.

^*^ *p*<0.05.

One individual did not have a scenario recorded.

Mean difference is calculated as pre–post.

A secondary analysis was conducted to examine if there were any differences in demographics and pre-simulation results between those who completed the post-simulation questions and those who did not. For the 364 health professionals who have gone through the simulation, there were no differences on demographics or pre-training questions between the 158 (43.4%) who completed both pre- and the post-simulation questions and the 206 that completed either the pre- or the post-simulation questions. However, ∼45.0% (*n*=164) of participants completed the post-simulation questions.

There were some slight differences between those who completed the post-simulation questions and those who did not. Those who completed the post-simulation questions were less likely to work in a federally qualified health center compared with those who did not complete the post-simulation questions (34.8% vs. 52.5%, *p*=0.03).

Those who completed the post-simulation questions were more likely to have a practice that did not see Medicaid patients compared with those who did not complete the post-simulation questions (33.9% vs. 17.5%, *p*=0.008). There were differences in responses to two of the pre-simulation questions. Participants that did not complete the post-simulation questions felt more strongly that the patient is largely responsible for being in their current circumstances compared with those who completed the post-simulation questions (2.15 vs. 1.91, *p*=0.04). Additionally, there was a slight difference in attempting to act in nonprejudiced ways with those who complete the post-simulation questions having a slightly higher mean compared with those who did not complete the post-simulation questions (4.69 vs. 4.51, *p*=0.04) (data not shown).

## Discussion

Completion of this interactive simulation in which a participant is given a window into the life course and experience of a patient for whom one may have negative biases resulted in somewhat less negative emotional and attitudinal responses to post-simulation survey questions as compared with pre-simulation responses. As the simulation aimed to capitalize on the individuation and perspective-taking components of evidence-based prejudice habit-breaking interventions,^8,9^ it appeared that learning more about the patient's experiences resulted in more compassionate feelings and thoughts about the patient. The pre- to post-simulation increase in frustration may be due to increased awareness of the barriers encountered by the simulation characters.

Participation in one training simulation cannot be assumed to modify health professionals' attitudes or behavior toward real patients, and any such impacts were not measured as part of this project. The goal of the effort was to develop a simulation informed by evidence-based theory (i.e., prejudice habit-breaking interventions) that could be used for training purposes. This simulation can be used as an introductory activity before further discussion about biases and their consequences.

Given that implicit biases contribute to disparities in health care,^5,6^ efforts to minimize the impact of biases represent a step toward health equity. Research with the prejudice habit-breaking model has demonstrated that opportunities for interaction with individuals from groups for which there are negative assumptions and biases can help mitigate the tendency to “fill in the blanks” with stereotypes for such individuals.^9,10^

Although training simulations cannot fully replace real-life experience with individuals from disenfranchised groups, they can begin to heighten awareness of how learning more of the history and life story for given individuals can help reduce negative thoughts and increase compassion for these persons. The more another person is viewed as an individual, rather than simply a member of a stereotyped group, the greater the chance that health care provided will be more equitable. Because habit development requires repetition, multiple simulation training activities and practice in real clinical settings is likely necessary for there to be consistent behavior change on the part of health professionals.

The Wright State University team is continuing its work with such training initiatives as additional funding has been received from the Ohio Department of Medicaid for the development simulations that address biases toward LGBTQ+ individuals and autistic persons. Input from individuals with lived experience is being prioritized in these new simulations in the interest of highlighting most relevant biases and advancing best practices.

### Limitations

Data reported in this study are from individuals who voluntarily completed the simulation, and provided responses to both the pre-and post-training questions. There is no expectation that the sample is representative of the general population of clinical and nonclinical health professionals. Also, pre- and post-simulation changes in survey responses cannot be generalized as predictive of attitudinal or behavioral changes in interactions with patients for whom an individual may have negative biases.

Ideally, the use of simulations in training will help prompt health professionals to modify their behavior with patients (e.g., inhibit the tendency to “fill in the gaps” with stereotyped assumptions), and as more equitable and inclusive habits are developed, manifestations of biases are less prominent. Future research should obtain measures of biases and behavioral manifestations of biases before and subsequent to involvement in training activities that include simulations such as ours to ascertain the actual effectiveness of such training in the clinical setting, and preferably would include repeated measures over a period of time.

### Conclusion

This engaging exercise represents the exciting interface of health professions' education and VR in a manner that allows the training experience to be completed remotely using one's own device. Use of the simulation to date suggests that it can be a meaningful component of initiatives to increase the cultural proficiency of individuals in health care professions. The simulation is available online so that health care organizations that desire to use is as part of staff training (e.g., diversity, equity, and inclusion) can readily do so. One possibility is to have participants complete the simulation and follow that with discussion and further programming on the topic of biases and health equity.

Your virtual patient is waiting to be seen! Initiating the simulation simply involves going to vpi.wright.edu or having one's device reader center on the following QR code: 
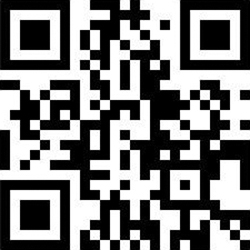


## Abbreviations Used

MEDTAPP: Medicaid Technical Assistance and Policy Program
MES: Medicaid Equity Simulation
SD: standard deviation
SMDs: standardized mean differences
VR: virtual reality

## References

[1] Kahneman D. Thinking, Fast and Slow. New York, NY: Farrar, Straus, Giroux, 2011.

[2] Roberts SO, Rizzo MT. The psychology of American racism. Am Psychol. 2021;76:475–487. 32584061 10.1037/amp0000642

[3] Hooper N, Erdogan A, Keen G, et al. Perspective taking reduces the fundamental attribution error. J Context Behav Sci. 2015;4:69–72.

[4] Hall WJ, Chapman MV, Lee KM, et al. Implicit racial/ethnic bias among health care professionals and its influence on health care outcomes: a systematic review. Am J Public Health. 2015;105:e60–e76. 10.2105/AJPH.2015.302903 PMC4638275 26469668

[5] FitzGerald C, Hurst S. Implicit bias in healthcare professionals: a systematic review. BMC Med Ethics. 2017;18:19. 28249596 10.1186/s12910-017-0179-8 PMC5333436

[6] Dovidio JF, Fiske ST. Under the radar: how unexamined biases in decision-making processes in clinical interactions can contribute to health care disparities. Am J Public Health. 2012;102:945–952. 22420809 10.2105/AJPH.2011.300601 PMC3483919

[7] Devine PG. Stereotypes and prejudice: their automatic and controlled components. J Pers Soc Psychol. 1989;56:5–18.

[8] Forscher PS, Mitamura C, Dix EL, et al. Breaking the prejudice habit: mechanisms, timecourse, and longevity. J Exp Soc Psychol. 2017;72:133–146. 29225371 10.1016/j.jesp.2017.04.009 PMC5720145

[9] Devine PG, Forscher PS, Austin AJ, et al. Long-term reduction in implicit race bias: a prejudice habit-breaking intervention. J Exp Soc Psychol. 2012;48:1267–1278. 23524616 10.1016/j.jesp.2012.06.003 PMC3603687

[10] Burns MD, Monteith MJ, Parker LR. Training away bias: the differential effects of counterstereotype training and self-regulation on stereotype activation and application. J Exp Soc Psychol. 2017;73:97–110.

[11] Chapman EN, Kaatz A, Carnes M. Physicians and implicit bias: how doctors may unwittingly perpetuate health care disparities. J Gen Intern Med. 2013;28:1504–1510. 23576243 10.1007/s11606-013-2441-1 PMC3797360

[12] Chapman MV, Hall WJ, Lee K, et al. Making a difference in medical trainees' attitudes toward Latino patients: a pilot study of an intervention to modify implicit and explicit attitudes. Soc Sci Med. 2018;199:202–208. 28532893 10.1016/j.socscimed.2017.05.013 PMC5714690

[13] Stone J, Moskowitz GB, Zestcott CA, et al. Testing active learning workshops for reducing implicit stereotyping of Hispanics by majority and minority group medical students. Stigma Health. 2020;5:94–103. 33134507 10.1037/sah0000179 PMC7597671

